# Procedural safety of transcatheter aortic valve replacement with Portico valve: a systematic review

**DOI:** 10.1097/JS9.0000000000000645

**Published:** 2023-08-15

**Authors:** Vikash Jaiswal, Zarghoona Wajid, Vinay Suresh, Muhammed Hanif, Kripa Rajak, Anupam Halder, Evbayekha Endurance, Henry Aiwuyo, Jinal Choudhari, Sidra Naz, Song P. Ang, Abhigan B. Shrestha

**Affiliations:** a Division of Cardiovascular Research, Larkin Community Hospital, South Miami, Florida; b The University of Texas, MD Anderson Cancer Center, Texas; c Department of Internal Medicine, Wayne State University School of Medicine, Michigan; d Department of Internal Medicine, SUNY Upstate Medical University, Syracuse, New York; e Department of Internal Medicine, UPMC Harrisburg, Pennsylvania; f Internal Medicine Department, St. Luke’s Hospital, St. Louis, Missouri; g Internal Medicine Department, St. Luke’s Hospital, St. Louis, Missouri; h Department of Medicine, King George’s Medical University, Lucknow; i Department of Medicine, Bangalore Medical College and Research Institute, Karnataka; j JCCR Cardiology Research, Varanasi, India; k Department of Internal Medicine, M Abdur Rahim Medical College, Dinajpur, Bangladesh

**Keywords:** aortic stenosis, outcomes, Portico valve implantation, transcatheter aortic valve replacement

## Abstract

**Background:**

The Portico transcatheter aortic heart valve is a self-expandable, fully resheathable bioprosthetic valve with a nitinol frame and porcine pericardial sealing cuff. It has been used among symptomatic severe aortic stenosis (AS) who are at high or extreme surgical risk. However, till date very few studies has been reported with inconclusive evidence for its postprocedure safety outcomes.

**Objective:**

The authors aim to evaluate the safety of the Portico transcatheter aortic valve replacement system among patients with AS.

**Methodology:**

The authors conducted a systematic literature search on PubMed, Embase, and Scopus from inception till 10th April 2023 by using predefined MESH terms using ‘AND’ and ‘OR’. The following search terms were used: ‘Aortic Stenosis’ AND ‘Transcatheter aortic valve replacement’ OR ‘Portico valve’. Finally, descriptive statistics were used to summarize the data in this paper. The mean and SD were adopted to describe continuous variables, whereas frequencies and percentages were used for dichotomous data.

**Results:**

A total of 7 studies with 2782 patients were included in the analysis. The mean age of patients was 82.3 years, and 54.63% were female. The most common comorbidity was hypertension (65.21%) and diabetes mellitus (26.45%). Among patients of AS with Portico valve implants, postprocedural outcomes including 30-day mortality (2.32%), cardiovascular mortality (2.37%), stroke (2.23%), myocardial infarction (0.94%), major bleeding (3.97%), major vascular complications (4.91%), acute kidney injury (1.37%), and permanent pacemaker implantations in 15.73% patients were reported. Overall, device success was observed in 95.82% of patients.

**Conclusion:**

Transcatheter aortic valve replacement with the repositionable Portico valve, a new bioprosthesis, appears to have a low postprocedural mortality rate and other clinical outcomes in high-risk patients with severe AS.

## Introduction

Highlights

- The transcatheter aortic valve is recommended to significantly reduce the mortality and symptoms among patients with severe symptomatic aortic stenosis. However, no systematic analysis has been done to summarize all the limited literature evaluating the safety of Portico valves.
- This very first systematic review shows that transcatheter aortic valve replacement with the Portico valve appears to be safe and effective with low mortality and clinical outcomes in high-risk patients with severe aortic stenosis.
- Most studies do not have long-term follow-up data with echocardiographic outcomes, encouraging further research and comparison with different valve types.

Transcatheter aortic valve implantation (TAVI) is a minimally invasive procedure that offers a less invasive approach to replace a dysfunctional aortic valve, commonly due to severe aortic stenosis (AS). TAVI has emerged as a well-established and effective treatment option for patients with severe AS who are at high surgical risk. Due to its minimally invasive nature, TAVI offers a safe alternative to surgical aortic valve replacement, with reduced death and disability as evidenced by the PARTNER 1 and PARTNER 2 trials^1,2^. TAVI has significantly evolved since its first introduction in 2002, with newer and innovative devices being developed and refined over the years. The Portico device is one of the latest TAVI devices to enter the market and has shown promise in clinical trials. It was first awarded the CE Mark in 2012 and received FDA approval in 2021 for use with the FlexNav Delivery System^3^. It is a self-expandable bioprosthetic, self-expanding and repositionable valve system made up of three bovine pericardial leaflets and a nitinol frame, as well as a porcine pericardial sealing cuff. Its outflow stent frame is designed with retention tabs that secure the crimped valves during deployment. The device is available in four sizes based on inflow measurements: 23 mm, 25 mm, 27 mm, and 29 mm and is typically inserted through transfemoral or trans-subclavian routes using a delivery catheter. The catheter is equipped with a soft, tapered nose cone, and an 18 Fr capsule that contains the compressed valve^4^. Overall, the Portico device offers a unique and innovative approach to TAVI that has shown promising outcomes in clinical trials^5,6^. The advantages of the Portico valve are its flexibility and low profile, making it easier to use in potentially challenging cases of torturous, calcified vessels^7^. Furthermore, retrieving and repositioning the Portico valve adds to the advantageous feature of this valve system^4^.

Despite promising outcomes in clinical trials, there have been limited studies with the Portico device and conflicting results regarding the incidence of postoperative complications and permanent pacemaker implantation (PPI)^8,9^. Thus, we sought to perform a systematic review to describe short-term safety and clinical outcomes post-TAVR by using the Portico valve.

## Methods

This systematic review was conducted and reported in conformity with the Cochrane and PRISMA (Preferred reporting items for systematic review and Meta-analysis), Supplemental Digital Content 1, http://links.lww.com/JS9/A889, Supplemental Digital Content 2, http://links.lww.com/JS9/A890 2020 and AMSTAR (Assessing the methodological quality of systematic reviews), Supplemental Digital Content 3, http://links.lww.com/JS9/A891 guidelines as described previously^10–12^. The prespecified protocol has been registered on Prospero (CRD42023411524).

### Search strategy

We conducted a systematic literature search in MEDLINE, Embase, Scopus, Web of Science using predefined MESH terms by using ʻANDʼ and ʻORʼ. The following search terms were used: (((((((Aortic Stenosis [MeSH Terms]) OR (TAVI[OtherTerm])) OR (Transcatheter aortic valve replacement [OtherTerm])) AND (Portico Valve[Other Terms])) OR (valve [Other Term])) AND (outcomes[Other Term]) OR (Mortality[Other Term]) OR (Stroke [Other Term]).

### Eligibility criteria

Studies were included if they fulfilled the following criteria: patients 18 years of age with a definitive diagnosis of AS, studies with Portico Valve use, and postimplantation safety outcomes. Studies such as prospective and retrospective were sought to be eligible. Studies that involved animal testing, any other valve type, no desired outcomes, and case report, case series, and review articles were excluded.

### Study selection

We queried databases from inception till 10th April 2023 without language restriction. The studies were carefully screened and exported to the Endnote 2020 library (X9). Two reviewers (J.C. and V.J.) reviewed the titles and abstract. Discrepancies regarding the inclusion of studies were arbitrated by the senior author (J.W.). The same reviewers also performed the full-text screening independently to decide which articles fulfilled the inclusion criteria. The senior author arbitrated discrepancies regarding the inclusion of studies.

### Data extraction and statistical analysis

The following data were extracted from the studies: demographic data (age and sex), study design, publication year, patient comorbidities, complications, and outcomes. Two authors (J.C., H.A.) assembled all available information in a shared Excel 2019 spreadsheet. For missing, incorrect, or unreported data, the corresponding authors of the respective papers were contacted via e-mail for clarification. Supplementary material related to the main article was also investigated in such cases. Finally, descriptive statistics were used to summarize the data in this paper. The mean and SD were adopted to describe continuous variables, whereas frequencies and percentages were used for dichotomous data. Two investigators (S.N. and K.R.) independently appraised the potential risk of bias using the Newcastle–Ottawa (NOS) scale for observational studies. We then classified studies as low, moderate, or high quality based on the scores after evaluation. All statistical analyses were conducted using the software R (v4.1.2; R Core Team 2023) (available at https://www.R-project.org/).

## Results

### Study selection

**Figure 1:**
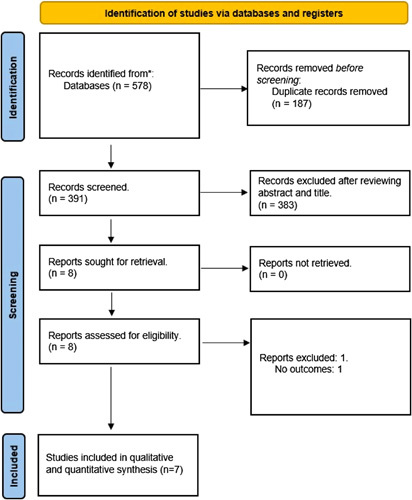
Preferred Reporting Items for Systematic Review and Meta-analysis flow of the search strategy for systematic review and meta-analysis.

The preliminary search using a predetermined search strategy yielded 578 articles, of which 187 studies were excluded as duplicates. Three hundred eighty three articles were subsequently excluded through title and abstract screening based on the inclusion criteria determined. A full-text review was done for the remaining nine studies identified during the search period. Furthermore, two studies were removed as they had no outcomes or were review articles. Finally, seven studies were included in the review, of which all are clinical cohort-based studies conducted between 2011 and 2022. The Preferred Reporting Items for Systematic Reviews and Meta-Analyses (PRISMA) flow diagram is depicted in Figure 1. The quality assessment of the observational studies was a low risk of bias on NOS for all observational studies. (Supplementary Table 1, Supplemental Digital Content 4, http://links.lww.com/JS9/A892).

### Baseline characteristics of the included patients

A total of seven^3,4,8,13–15^ studies with 2782 patients were included in this systematic review. The mean age of the patients was 82.3 years, with 1520 (54.63%) female patients. All patients included were diagnosed with severe symptomatic AS. The STS (Society of Thoracic Surgeons) Score was calculated in five studies, with a mean value of 6.08±1.6. The most common comorbidity include hypertension in 65.21% (*n*=1663/2550) patients, diabetes mellitus in 26.45% (*n*=736/2782) patients, followed by, myocardial infarction in 9.63% (*n*=267/2772) of patients, 6.42% (*n*=178/2772) having a past history of stroke, 10.19% (*n*=272/2668) with history of peripheral artery disease, and 31.94% (*n*=849/2658) with history of atrial fibrillation. 23.92% (*n*=636/2658) patients underwent percutaneous coronary intervention (PCI), and 11.58% (*n*=309/2668) patients had PPI. The mean LVEF was 51.04±15.76 while the mean aortic valve area (AVA) was 0.67±0.04. The mean transaortic gradient (in mmHg) recorded was 44.26±2.6. Moderate to severe mitral regurgitation was present in 5.55% (*n*=139/2503) of patients (Table 1).

**Table 1:** Baseline characteristics of patients included in the studies.

Variables	Blumenstein *et al*., 2022^8^	Corcione *et al*., 2020^13^	Perlman *et al*.^4^	Willson *et al*.^14^	Mollmann *et al*.^3^ (PorticoTM DS)	Mollmann *et al*.^3^ (FlexNavTM DS)	Möllmann *et al*.^15^	Raj R Makkar *et al*.
Sample size	344	114	57	10	501	500	222	1034
Study design	Nonrandomized cohort	Observational cohort	Observational cohort	Observational cohort	Observational cohort	Observational cohort	Prospective, nonrandomized, multicenter study	RCT
Age, years (Mean)	82.92	82.4	80.8	82.4	81.7	82.3	83.0	83±7
Female, *n* (%)	206 (59.9)	70 (61)	47 (82.5)	10 (100%)	320 (63.70%)	307 (61.40%)	165 (74.32%)	395 (52·7%)
NYHA Class III/IV	290	85/1	43	8/0	295 / 32	293/18	166/ 9	229 (60·1%)/43 (11·3%)
STS score	–	–	7.7±5.7	8.1 3.2	4.2±2.9	4.2±2.7	5.8±3.3	6·5 ±3·4
Diabetes, *n* (%)	120	21	20	5	176	182	69	143 (37·5%)
Hypertension, *n* (%)	308	80	46	–	440	431	–	358 (94·0%)
Previous MI, *n* (%)	37	10	10	–	68	61	26	55 (14·4%)
Previous stroke, *n* (%)	31	2	7	–	53	38	18	29 (7·6%)
Previous peripheral artery disease, *n* (%)	48	–	14	1	60	59	18	72 (18·9%)
Previous atrial fibrillation, *n* (%)	135	–	20	–	246	238	85	125 (32·8%)
Mean AVA, cm^2^	–	0.6	0.7	0.62	0.71	0.72	–	0·68±0·17
LVEF,%	59.25	55	58.4	57.3	–	–	>20	57·3 (11·5)
Moderate to severe mitral regurgitation, n	5	50	–	5	1	1	–	78 (20·5%)
Mean Transaortic gradient, mmHg	42.75	50	41.8	44.5	43.4	42.2	43.3	46·2 (11·2)

### Postprocedural outcomes and device success

Among patients of AS with Portico valve, postprocedural outcomes including 30-day mortality (2.32%, *n*=62/2668), in-hospital mortality (0.75%, *n*=11/1459), cardiovascular mortality (2.37%, *n*=29/1223), stroke (2.23%, *n*=61/2725), myocardial infarction (0.94%, *n*=16/1691), major bleeding (3.97%, *n*=108/2715), major vascular complications (4.91%, *n*=134/2725), acute kidney injury (1.37%, *n*=36/2611), PPIs (15.73%, *n*=266/1691), and 0.36% (*n*=6/1624) patients had coronary obstruction after the procedure. Overall device success was seen in 95.82% (*n*=1675/1748) patients, 0.245% (*n*=3/1223) patients reported to have an annular rupture, and 1.78% (*n*=6/336) patients reported undergoing valve-in-valve procedures (Table 2).

**Table 2:** Clinical outcomes postprocedure, and at follow-up post-Portico valve implantation.

Variables	Blumenstein *et al*., 2022^8^	Corcione *et al*., 2020^13^	Perlman *et al*.^4^	Willson *et al*.^14^	Mollmann^3^ (PorticoTM DS)	Mollmann^3^ (FlexNavTM DS)	Möllman *et al*.^15^	Raj R Makkar *et al*.
Sample size	344	114	57	10	501	500	222	1034
All-cause mortality, *n*	–	16	9	–	–	10	–	53 (14·3%)
In-hospital mortality, *n*	10	0	–	–	1	0	–	–
Cardiovascular Mortality^a^, *n*	–	–	–	–	15	6	8	–
30 Days mortality, *n*	13	–	2	0	16	10	8	13 (3·5%)
Stroke^a^, *n* (in hospital/ follow-up)	13/–	0/0	–/4	1/–	13/–	16/–	12/–	6
MI^a^, n (in hospital/ follow-up)	6/–	0/0	–/1	0/–	2/–	1/–	7/–	–
Major bleeding^a^, *n* (in hospital/ follow-up)	2/–	0/0	–/4	–	26/–	33/–	25/–	22
Major vascular complications^a^, *n* (in hospital/ follow-up)	9/–	0/0	–/5	0/–	32/–	41/–	16/–	36
AKI^a^, *n* (in hospital/ follow-up)	12/–	–	–/1	0/–	7/–	4/–	9/–	4
PPI^a^, *n* (in hospital/ follow-up)	52/–	13/14	–/5	0/–	87/–	84/–	30/–	–
Coronary Obstruction, *n*	0	–	0	–	2	3	1	–
Major adverse event^a^, *n* (in hospital/ follow-up)	–	0/16	–	–	–	–	–	–
Device success, *n*	318	112	43	10	488	488	216	–
Annular rupture, *n*	–	–	–	–	2	1	0	–
Valve-in-valve procedure, *n*	–	2	–	–	0	0	4	–
AVA, cm^2^ postprocedure	–	1.8 (2.2)	1.27±0.31	1.3±0.2	–	–	1.9±0.5	1·85±0·46

a Data arranged in form of postprocedure outcomes/outcomes at follow-up.

## Discussion

**Figure 2:**
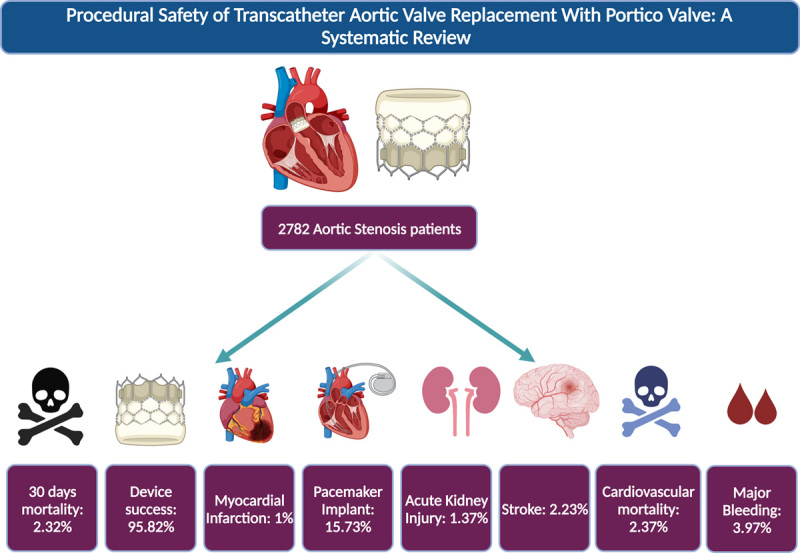
Central illustration highlighting the clinical outcomes post-Portico valve implantation.

To our knowledge, this is the very first and most comprehensive systematic review to date, with the highest sample size evaluating the clinical outcomes after transcatheter valve implantation with a Portico valve. In our study, postimplantation 30 days mortality was 2.32%, less than reported by Linke *et al*. (3.6%) and Perlman *et al*. (3.5%)^4,16^. Furthermore, our analysis showed that the 30-day-mortality due to cardiovascular causes was 2.37%, which was also less than reported by Linke *et al*. (3.6%)^16^. Our study reported post-Portico valve implantation in-hospital mortality was 0.75%, stroke was 2.23%, myocardial infarction was 0.94%, major bleeding was 3.97%, the major vascular complications were 4.91%, and acute kidney injury was 1.37% (Fig. 2).

The new generation devices such as Portico and Evolut were compared by Giordano *et al*. and Corcione *et al*., and they reported that no significant differences were found in procedural and in-hospital stroke, myocardial infarction, major bleeding, major vascular complications, and all-cause mortality^13,17^. However, follow-up data by Giordano *et al*. showed significantly higher all-cause mortality (14.3 vs. 5.3%) and major adverse events (14.3 vs. 5.3%) post-Evolut interventions in comparison to those patients who underwent transcatheter valve implantation with the Portico valve^17^. Similarly, Evolut was associated with significantly higher pacemaker implantations and lower peak and mean aortic gradients compared to the Portico valve^17^.

All five devices (Portico, Evolut, Acurat, Lotus, and Sapien3) were compared by Giordano *et al*.^18^, and they reported that the Portico valve was associated with significantly reduced major adverse events, major vascular complications, renal failure, and pericardial effusion in comparison to the other four-valve devices. However, no significant differences were found in terms of all-cause mortality, cardiovascular death, stroke, and myocardial infarction among all five devices^18^. Another study conducted by Trigo *et al*.^19^ compared the Portico valve with Sapien XT and found that the Portico valve was associated with a lower rate of myocardial infarction, pacemaker implantation, and conversion to open heart surgery while a higher rate of stroke and major bleeding, although the result was nonsignificant (*P*>0.05). Blumenstein *et al*.^8^ compared the Portico valve with the Acurate Neo and no significant differences were reported in terms of stroke, major bleeding, myocardial infarction, renal failure, and in-hospital mortality in both the groups. They reported rare instances of urgent conversion to sternotomy, which was prompted by complications such as coronary impairment, THV embolization, pericardial effusion, and severe mitral regurgitation due to wire perforation. Willson *et al*.^14^ demonstrated successful recapture and repositioning of the Portico valve in four cases of suboptimal implantation. Similarly, Möllmann *et al*.^15^ demonstrated successful valve resheathing and repositioning in one third of the procedures to address suboptimal implantation depth and paravalvular leakage. The study conducted by Mollman H *et al*.^3^ revealed instances of unsuccessful valve implantation, resulting in the use of alternative nonstudy valves. Additionally, cases of annular rupture, coronary obstruction, and the requirement for a secondary valve within 30 days were documented^3^. Notably, among the subjects, two individuals (0.2%, 2/1001) necessitated a second valve due to paravalvular leakage^3^. One subject received a second Portico valve, while the other subject underwent implantation with a nonstudy valve^3^.

The Portico Valve consists of a self-expandable frame with a pericardial sealing cuff and three pericardial leaflets^20^. It has large cells with more tissue and less metal that can conform better around the native valve leaflets, improving valve apposition and reducing the risk of leaks from the aorta to the ventricle^21^. The valve is fully repositionable and retrievable and indicated for patients with high-risk for open-heart surgery and hemodynamically unstable patients during the procedure^21^. Recently the FDA approved this valve for use in severe AS patients at high-risk for SAVR^22^. However, this procedure is not risk-free, and life-threatening bleeding, acute renal injury, stroke, the need for permanent pacemaker replacement, and death has been reported in the literature^23^.

The Portico valve is the choice of valve for patients having large annulus size (>27 mm), in patients having severe aortic calcifications, in patients having a bicuspid aortic valve, and in patients using small vessels as access routes of entry^24^. The leaflet material used for Portico valve is of bovine tissue as compared to Evolut and ACCURATE, which are made up of porcine tissue^24^. The Portico valve and Evolut valves are repositionable, retrievable, and resheathable while SAPIEN 3 and ACCURATE valves are not repositionable and retrievable^24^. The access routes for Portico valve are transvascular and transaortic with an 18 or 19 Fr delivery system, while for SAPIEN access routes are transvascular, transaortic, and transapical with 14–16 Fr inner diameter of sheath, for Evolut R and PRO access routes are transvascular and transaortic with an inline sheath diameter of 14 Fr, and for ACCURATE neo access routes are transapical and transvascular with a 14 Fr inner diameter of the sheath^24^. Recently, with the introduction of the Newer FlexNav delivery system, the results are more promising and have further reduced all-cause mortality, cardiovascular mortality, acute kidney injuries, myocardial infarction events, and new PPIs as compared to the old delivery system of the Portico valve^3^. However, a large trial needs to be conducted on these newer FlexNav delivery systems compared to the older delivery system and other valve types to get more significant results.

### Limitations

This review includes only observational studies, so they are subject to publication bias. Secondly, most studies did not have reported long-term follow-up data, which can be something to research further and evaluate the outcomes. Lastly, we cannot evaluate the incidence rate because of the limited number of events and study sample size.

## Conclusion

Transcatheter aortic valve replacement with the Portico valve appears safe and effective, with low mortality and clinical outcomes in high-risk patients with severe AS. However, most studies do not have long-term follow-up data, including echocardiographic outcomes, which encourage further research and comparison with different valve types.

## Ethical approval

Since this is a review article of previously published studies, hence ethical approval is not required.

## Consent

None.

## Sources of funding

None.

## Author contribution

V.J.: contributed to the conception or design of the work; V.J., V.S., Z.W.: contributed to the acquisition, analysis, or interpretation of data for the work; V.J., E.E., V.S., M.H., K.R., S.N.: drafted the manuscript. All authors critically revised the manuscript. All gave final approval and agreed to be accountable for all aspects of work ensuring integrity and accuracy.

## Conflicts of interest disclosure

V.J. serves as an Associate Editor in the European Journal of Medical Research in Cardiology section (Unpaid).

## Research registration unique identifying number (UIN)

This paper was registered in Prospero registration: CRD42023411524.

## Guarantor

Vikash Jaiswal.

## Data availability statement

The data underlying this article are available in the article and its online supplementary material.

## Provenance and peer review

Not commissioned, externally peer reviewed.

## Supplementary Material
