# Supplementary figures and images for: Procedural safety of transcatheter aortic valve replacement with Portico valve: a systematic review
Source: Int J Surg. 2023 Aug 15;109(11):3602–8. doi: 10.1097/JS9.0000000000000645 (PMC10651300; doi:10.1097/JS9.0000000000000645)

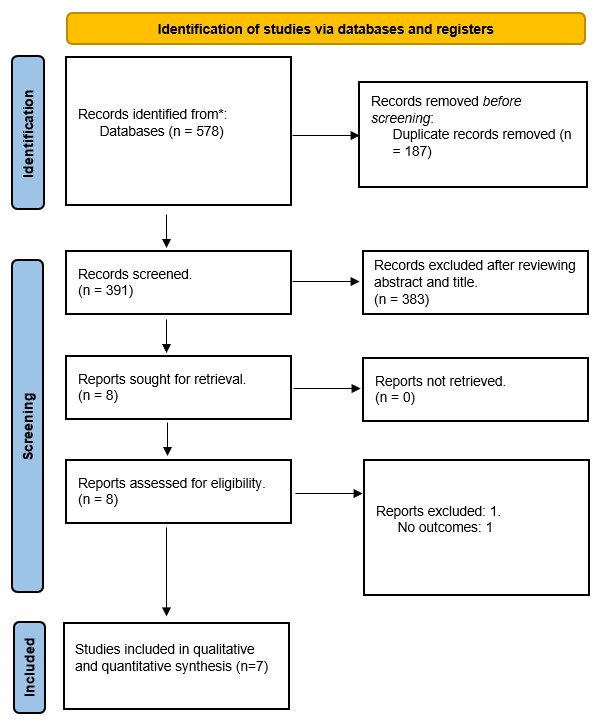

Supplement: Supplementary file 2 [file js9-109-3602-s002.docx]
