# Supplementary material for: Procedural safety of transcatheter aortic valve replacement with Portico valve: a systematic review
Source: Int J Surg. 2023 Aug 15;109(11):3602–8. doi: 10.1097/JS9.0000000000000645 (PMC10651300; doi:10.1097/JS9.0000000000000645)
Supplement: Supplementary file 4 [file js9-109-3602-s004.docx]

**Supplementary Table 1:** Newcastle-Ottawa scale for quality assessment and bias assessment of observational studies.

| Study | Selection | | | Comparability | | Outcome | | | Total* |
| --- | --- | --- | --- | --- | --- | --- | --- | --- | --- |
| Author, year | Representativees of exposed group | Selection of non-exposed cohort | Ascertainment of exposure | Outcome of interest |  | Outcome assessment | Adequacy of follow-up duration | Adequacy of follow-up of cohort |  |
| Blumenstein et al | 1 | 1 | 1 | 1 | 2 | 1 | 0 | 0 | 7 |
| Corcione et al | 1 | 1 | 1 | 1 | 2 | 1 | 1 | 1 | 9 |
| Perlman et al | 1 | 1 | 1 | 1 | 2 | 1 | 1 | 1 | 9 |
| Willson, et al | 1 | 1 | 1 | 1 | 2 | 1 | 0 | 0 | 7 |
| Mollmann et al (PorticoTM DS) | 1 | 1 | 1 | 1 | 2 | 1 | 0 | 0 | 7 |
| Mollmann et al (FlexNavTM DS) | 1 | 1 | 1 | 1 | 2 | 1 | 0 | 0 | 7 |
| Mollmann et al | 1 | 1 | 1 | 1 | 2 | 1 | 0 | 0 | 7 |
| Raj R Makkar et al | 1 | 1 | 1 | 1 | 2 | 1 | 1 | 0 | 8 |
